# Supply-demand balance and spatial distribution optimization of primary care facilities in highland cities from a resilience perspective: A study of Lhasa, China

**DOI:** 10.3389/fpubh.2023.1131895

**Published:** 2023-03-08

**Authors:** Yang Yu, Rui Zhou, Liyuan Qian, Xian Yang, Liuyang Dong, Guangyuan Zhang

**Affiliations:** ^1^School of Architecture, Southwest Jiaotong University, Chengdu, China; ^2^School of Transportation and Logistics, Southwest Jiaotong University, Chengdu, China

**Keywords:** resilient city, public health, spatial analysis, urban planning, highland area

## Abstract

**Introduction:**

The development of urban resilience, which is fundamentally a balance between the supply capacity of primary care resources and the demand from urban residents, includes an appropriate architecture of primary care facilities. Resilient city construction in highland areas is hampered by the physical environment and transportation constraints and frequently encounters issues like poor accessibility and unequal distribution of primary care facilities.

**Methods:**

To optimize the supply and demand of primary care resources in highland cities and effectively improve the resilience of urban public health, this paper assesses the distribution of primary care facilities within the built-up area of Lhasa (China) through a spatial network analysis method based on GIS, combined with population distribution data, and employs a location-allocation model to optimize the distribution.

**Results:**

Firstly, the overall supply of primary care exceeds the overall demand, but the facilities' service area can only accommodate 59% of the residences. Secondly, there is a clear spatial variation in the accessibility of primary care facilities, and the time cost of healthcare is too high in some residences. Thirdly, the supply-demand relationship of primary care facilities is unbalanced, with both over-saturated and over-deficient areas.

**Discussion:**

After distribution optimization, the coverage and accessibility of primary care facilities have increased significantly, and the spatial imbalance of supply and demand has been alleviated. This paper proposes a research method to evaluate and optimize the spatial distribution of primary care facilities from multiple perspectives based on the resilience theory. The results of the study and visualization analysis methods can be used as an invaluable reference for planning the distribution of urban healthcare facilities and urban resilience construction in highland areas and other underdeveloped areas.

## Introduction

Widely employed in economic (1, 2), social (3), ecological (4, 5), and urban safety (6, 7) disciplines, the idea of resilience refers to the capacity of a system, community, or city to resist, absorb, adapt, and recover from hazard consequences in a timely and effective manner (8–10). Introducing the notion of resilience into urban planning can help cities effectively withstand the effects of risks such as climate change, natural disasters, and resource depletion and improve the stability of urban operations and capacity for comprehensive catastrophe prevention (11–13). Since 2019, as a result of the COVID-19 pandemic, the level of urban public health construction and services has now become an essential criterion for gauging the resilience of cities (14–18). Improving urban public health systems and reducing vulnerability and increasing the resilience of health systems have become major goals of national health policies (19–21). In this context, how to optimize the healthcare service system and increase public health resilience is one of the most important directions for advancing urban resilience theory (22).

The majority of the healthcare service system consists of hospitals, primary care facilities, and specialized public health facilities. Among them, primary care facilities serve as the primary providers of community medicine, infectious disease prevention, and healthcare. The Declaration of Astana proclaims, “We will benefit from sustainable public healthcare that enhances health systems resilience to prevent, detect and respond to infectious diseases and outbreaks” (23). Other previous studies have also linked primary care to public health resilience, demonstrating the positive effect of the primary care level on public health and the efficiency of the healthcare system (24–26). Areas with well-equipped primary care facilities also have healthier populations (27). Therefore, the rationality of the spatial distribution of primary care facilities is not only significant to the construction of urban public health resilience but also directly related to social equity (28–30). Compared to plain areas, cities in highland areas are confined by a severe natural environment and transportation limitations, making the cross-regional deployment of medical resources more challenging. As a result, primary healthcare facilities must shoulder more pressure to provide medical services. Moreover, most of these cities are situated in high-altitude and alpine regions, as well as underdeveloped areas, and the overall level of urbanization is low. Consequently, it is difficult to match the supply level of primary medical resources with the health needs of urban residents, and the spatial distribution has issues such as low accessibility and uneven distribution. However, previous studies on the distribution of healthcare facilities rarely address highland areas or focus on the supply-demand balance for primary care. Therefore, a comprehensive assessment of the supply and demand of primary healthcare resources in highland cities and optimization of the spatial distribution of primary care facilities are essential to improve the epidemic prevention and control capacity and the health level of residents in highland cities.

This paper uses the urban built-up area of Lhasa, China, as the study area. We use the resident population based on cell phone signaling data and the seventh national census data to calculate the resident medical demand and combine the network analysis method of the GIS platform to calculate the supply capacity of primary care facilities. On this basis, the rationality of the supply-demand relationship and spatial distribution of primary care facilities in Lhasa are evaluated in terms of redundancy, accessibility, and balance. An optimal distribution plan is then proposed using the locational allocation model, and the optimization results are validated. This paper's contributions can be summarized as follows. Firstly, it considers the distribution of population and the balance of supply and demand, since previous distribution evaluation studies are mostly from the perspective of facility accessibility. Secondly, previous studies on the optimal distribution of care facilities focus primarily on megacities or urban clusters with high economic development levels as the objects but less on cities in the underdeveloped areas of the highlands, which are complemented by this study. Thirdly, based on an evaluation of the supply-demand relationship and spatial distribution of primary care facilities, this study proposes an optimization scheme coupled with a locational allocation model that can not only be used as a guide for public health resilience construction and urban public service facilities planning but also be widely applied to the study and planning of primary care facilities in other highland areas and cities in underdeveloped regions.

The following sections comprise the remainder of this study. The “Literature Review” examines previous studies on urban resilience and the evaluation of primary care facilities. “Data” provides an overview of the research subject and data sources. “Methodology” outlines the geographic analytic techniques employed in this study. “Results” presents the results of the evaluation of the supply and demand relationship and spatial distribution of primary care facilities in Lhasa. “Discussion” discusses the “Results” section and proposes distribution optimization solutions. “Conclusion” highlights the study's key results, consequences, and limitations.

## Literature review

The concept of resilience has evolved from engineering resilience (31, 32) to ecological resilience (33) and then to evolutionary resilience (34, 35). After its introduction into the field of urban studies, urban resilience is defined as “the capacity to achieve normal functioning of public safety, social order, and economic construction by adequately preparing, buffering, and responding to uncertainty perturbations” (11, 36–40). Jha et al. (41) proposed four components of urban resilience based on this finding: infrastructure resilience, institutional resilience, economic resilience, and social resilience. Currently, urban resilience research focuses more on the maintenance and coordination capability of the social-ecological system (42–44). Public health resilience, as part of social resilience, is generally understood to be the ability of healthcare systems and facilities to maintain function and recover from public health crises, both sudden (e.g., natural disasters and COVID-19) and slow (e.g., chronic diseases, etc.) (45–47). Previous research on public health resilience can be split into two main categories. Some studies examine the benefits of individual healthcare facilities, taking into account factors like staff management, built environment, intelligent systems, and medical equipment (21, 48–51). The others broaden the focus to include urban healthcare networks, taking into account the quantity, distribution, and interactions of healthcare facilities (52–55). According to Kruk et al. (52) a resilient healthcare system should cover multiple levels of facilities with wide coverage, while Hassan and Mahmoud (54) argue that a resilient healthcare network should consider not only the quantity and quality of facilities but also the demand and accessibility of healthcare services to the population.

In urban resilience construction, community resilience plays a fundamental and crucial role (56). As the fundamental urban unit, the level of community resilience can influence a city's capacity to resist and recover from calamities. Therefore, primary care facilities serving the community are important components in terms of their large quantity and wide distribution. A reasonable distribution of primary care facilities can enhance the robustness of the healthcare system, the redundancy of healthcare resources, and the rapidity of residents' access to healthcare services (57, 58), thereby enhancing the resilience of urban public health (24, 26, 27, 59–61). Existing studies generally agree that the resilience of primary care facilities, which are the first point of contact between the population and the healthcare system (59), will be most severely tested in a pandemic such as COVID-19 (26). These facilities not only perform medical functions but also include prevention, immunization, rehabilitation, healthcare, and education throughout each period of the pandemic (60), which is consistent with the principles of the resilience concept: resistance, adaptation, and recovery. On this basis, Akman et al. (61) suggest that the capacity of primary care services can hardly develop spontaneously but needs to be well-organized and planned, which demands the optimization of the distribution of primary care facilities.

Existing studies on the location and arrangement of public facilities can be divided into two categories: distribution evaluation and distribution optimization (62–64). Among them, most distribution evaluation studies are conducted from the perspective of spatial accessibility to calculate the transportation convenience from residences to facilities to accurately identify the areas lacking public services due to poor accessibility and to provide distribution optimization recommendations (65–72). For instance, some scholars analyzed the accessibility of public playgrounds from the standpoint of spatial justice while taking numerous social potential aspects into account (67).

The study of optimization models is another crucial aspect of the distribution of public facilities research. According to the different considerations and optimization objectives, scholars in the disciplines of operations research, geography, and computational science have proposed numerous facility distribution optimization models, such as the P-median model that minimizes facility distance (73) and the coverage model that maximizes coverage area (74) in the traditional location model, the equity maximization model that pursues the minimum distance difference (75), the multi-objective model that minimizes the sum of all distance differences (76), and expanded models such as the dynamic locality model (77), stochastic locality model (78), and multi-level locality model (79), among others. Gu et al. (76) constructed a dual-objective planning model that takes into account the efficiency of distribution facilities and the maximum coverage area to explore the optimal distribution of preventive care facilities. Other scholars introduced the concept of service radius in the coverage model and combined it with a gravity model to identify areas lacking care facilities (80), etc. These models have been increasingly utilized in care facility distribution studies. These studies focused on the accessibility and service coverage of care facilities, with the accessibility studies seeking to minimize the access distance and improve the convenience of healthcare, and the service coverage studies seeking to maximize the coverage area to increase the supply capacity of medical services.

Most existing studies concentrate on Nanjing, Wuhan, Zhengzhou, and other large cities with favorable economic and social conditions, where the primary medical system is relatively complete, and the medical supply is sufficient. However, these cities have high population density and service pressure on primary care facilities. Consequently, spatial accessibility and convenience of facilities are regarded as important evaluation indices, while coverage capacity is considered less frequently. Studies on distribution optimization models concentrate on maximizing the service supply capacity of care facilities, with little attention paid to the effect of the geographical variation in the medical demand levels of residents. In contrast, the urban primary care system in highland areas is underdeveloped, and the ability to distribute resources among multi-level care facilities is limited. Simultaneously, the urbanization rate in these areas is lower. The topography restricts the geographical development of the city, and the spatial diversity of population density is more pronounced. Fully based on the characteristics of highland cities, this study combines the supply level of primary care facilities and the demand level of residents to evaluate the primary care services in Lhasa, China and optimizes the spatial arrangement of primary care facilities aiming for the supply-demand balance to make the results more objective and credible.

## Data

### Study area

As the capital of the Tibet Autonomous Region (China), Lhasa is the political, economic, cultural, and religious hub of Tibet. The city is in the midst of the Tibetan Plateau, in the valley plain of the Lhasa River (a tributary of the Yarlung Tsangpo River), at an elevation of 3,650 m above sea level. According to the seventh national census and Tibetan Statistical Yearbook, as of 2021, Lhasa has a population of 867,800 and a GDP of 67.8 billion yuan, ranking it last among China's provincial capitals in terms of economic volume.

**Figure 1:**
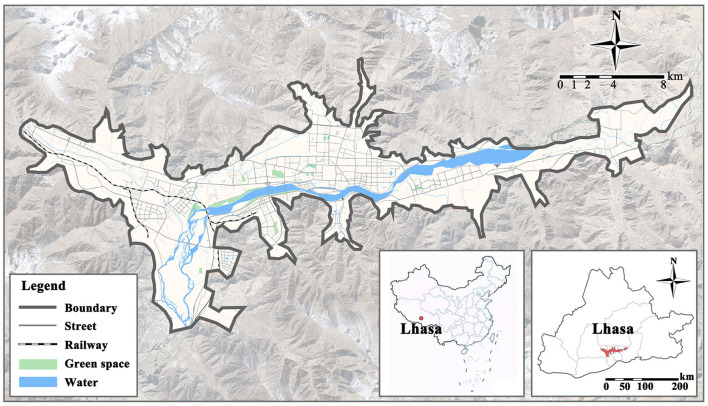
Map of the Lhasa study area.

This study identifies the urban built-up area under the jurisdiction of Lhasa by combining Landsat remote sensing imagery in 2021 (Figure 1), which covers portions of Chengguan, Dulongdeqing, and Dazi districts with a total size of 268.5 km^2^.

### Population distribution data

Existing statistics on urban populations typically use administrative districts or streets as the statistical unit, making it more challenging to capture the spatial distribution of the population to evaluate the amount of healthcare demand. With the advancement of information and communication technology, cell phone signaling, as a new type of data, can more accurately reflect the spatial distribution characteristics and spatiotemporal variation characteristics of the population and is thus widely used to study population distribution and activity characteristics (81, 82). In this study, we gathered the cell phone signaling data of Lhasa in September 2021 and coupled it with the city's seventh census data in the study area (Chengguan, Dulongdeqing, and Dartse districts) to acquire the final population distribution statistics. On this basis, this research splits the study area into 300 × 300 m grids using the network processing tool in GIS and then determines the population density in each grid. The formula is as follows.


(1) $$\begin{array}{l} {\text{P}}_{\text{i}}=\frac{{\text{P}}_{\text{T}}}{{\text{M}}_{\text{T}}}\times {\text{M}}_{\text{i}} \end{array}$$

Where **P**_**i**_ and **M**_**i**_ are the number of resident population and cell phone signaling in grid ***i***. **P**_**T**_ and **M**_**T**_ are the total number of the resident population and cell phone signaling in three urban districts, respectively. This conversion method can transform macro demographic data into geographic distribution data, thus clearly reflecting the amount of primary care demand.

### Facilities and residences distribution data

As an emerging data source, POI (Point of Interest) can depict the spatial distribution characteristics of residential areas and diverse service facilities (83, 84). In this paper, we used Python to crawl the POI data of Lhasa within the study area from Gaode Map (https://www.amap.com). We then selected the residential areas and villages as the residence data required for the study and the healthcare service category as the primary care facility data. Large general hospitals in the healthcare service category are also the main providers of medical services for residents. However, on the one hand, Lhasa is the capital city of the Tibet Autonomous Region. The 3-tier healthcare system in China consists of primary care facilities, secondary hospitals, and tertiary hospitals. The primary care system can be further divided into two categories: community clinics and health centers, with the former referring to community-based care facilities and the latter to subdistrict-based care facilities (85–87). The secondary and tertiary hospitals in the city must provide medical services to residents from other cities in the region at the same time, so it is difficult to accurately calculate the coverage area and healthcare supply of their service areas. On the other hand, the breadth of healthcare services provided by general hospitals and primary care facilities are not identical, and their positions in the development of public health resilience cannot be interchanged. As a result, these hospitals are only used as a reference for the research outcomes and not as the primary target of the investigation.

### Road network data

This study uses GIS spatial network analysis to investigate the relationship between the primary care facility supply-demand relationship and its spatial structure. A vector road network is required for precisely calculating the real geographic distance between care facilities and residences. Therefore, the vector road network in the Lhasa study area was downloaded from the OpenStreetMap platform (https://www.openstreetmap.org), and the road centerline data was extracted from it.

## Methodology

**Figure 2:**
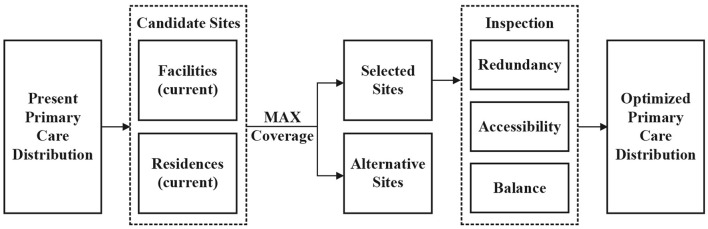
The framework for the impact of primary care facility distribution on urban public health resilience.

Combining the characteristics of urban resilience, this study evaluates the spatial distribution of primary care facilities in terms of redundancy, accessibility, and balance. These three aspects correspond to the redundancy, rapidity, and robustness of the famous resilience “4R” for physical and social systems (88), and play different roles in the three phases of urban resilience consisting of resisting, adapting, and recovering (89) (Figure 2). In addition, the resourcefulness in the “4Rs” requires a combination of redundancy, accessibility, and balance of primary care facilities to enhance the healthcare efficiency and the learning capacity of the system by enhancing the organizational capacity of the urban health system.

Firstly, the service area analysis method in GIS spatial network analysis is used to calculate the service coverage of primary care facilities and determine the overall supply and demand relationship. Secondly, the accessibility of residents to primary care facilities and their spatial differentiation characteristics are analyzed by constructing an Origin-Destination matrix and combining it with the space syntax method. Thirdly, the level of primary care demand is computed based on the study area's population. Finally, the level of primary care demand was computed using population distribution data and geographically overlapped with the level of medical supply to analyze the supply and demand balance of primary care facilities in Lhasa. Based on the assessment results, this paper combines the location-allocation model to optimize the spatial distribution of primary care facilities.

### Spatial analysis

This study's spatial analysis method is based on the GIS platform and consists of kernel density analysis, service area analysis, and Origin-Destination matrix analysis. Among them, kernel density analysis is used to calculate the spatial density of points in the surrounding search radius, which can reflect the distribution characteristics of primary care facilities and residences within the study area. Service area analysis and Origin-Destination matrix analysis are based on the network data set constructed by the GIS network analysis tool and can assess the service scope and accessibility of primary care facilities. In the examination of the service area, according to the requirements of the “15-min living circle” for service facilities, the service distance of community clinics and health centers are set at 5 and 15 min walking distance, i.e., 400 and 1,200 m, respectively (the walking speed is set at 80 m/min), which is used to calculate the service area coverage of care facilities. While the Origin-Destination matrix can be constructed, and the distance from the residential point to the closest care facility can be calculated. In addition, the service density within the service area of each facility level can be determined based on the number of people served and the size of the service area using the method shown below.


(2) $$\begin{array}{l} \text{D}=\frac{\text{P}}{\pi {\text{R}}^{2}} \end{array}$$

Where ***D*** is the service density (this study assumes that the service density is the same for each raster within a facility's service area). ***P*** is the number of people served by the facility, which is 5,000 for community clinics and 30,000 for the health center. ***R*** is the service distance.

### Space syntax

As an effective theory and method for spatial quantification, the computation of integration degree by space syntax may describe the spatial potential of the reached sites and is frequently used to reflect road accessibility, which is separated into global integration and local integration. The global integration represents the degree of connection between a node and all nodes in the region and can be used to reflect the overall accessibility of the distribution of care facilities in Lhasa. On the other hand, the local integration represents the degree of connection between a node and nodes within a few steps of the topology and can be used to reflect the degree of connection between each care facility location and its surrounding residential points. In this study, we use the axis analysis method in the Depthmap platform to transform the vector road network into at least the longest road axis, and we calculate and display the global integration and the local integration of the road axis to visually represent the spatial accessibility of each care facility.

### Location-allocation model

**Figure 3:**
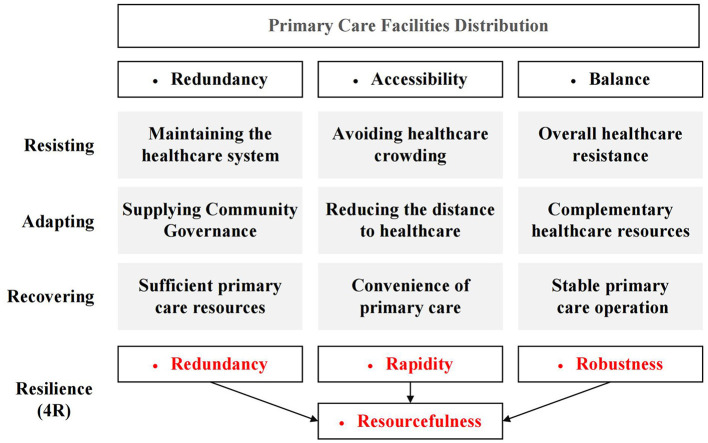
Maximized coverage model operation process.

The location-allocation model is a system that picks the facility site with the optimal service capacity from a large number of candidate facility locations based on the defined service demand and a specific optimization model to optimize facility architecture. The minimization facility point model, maximization coverage model, minimization impediment model, and maximization pedestrian flow model are the most prevalent location-allocation models today. Highland cities have limited medical resources and facility construction capacity, and it is difficult to optimize the spatial layout of facilities by simply increasing the healthcare supply. Therefore, the total quantity of primary care facilities before and after optimization is proposed to be unchanged in this study. The maximizing coverage model is used to calculate the optimal architecture of primary care facilities that maximize coverage of current residential sites by considering existing primary care facility sites and residential sites as candidate facility sites, as illustrated in Figure 3.

## Results

**Figure 4:**
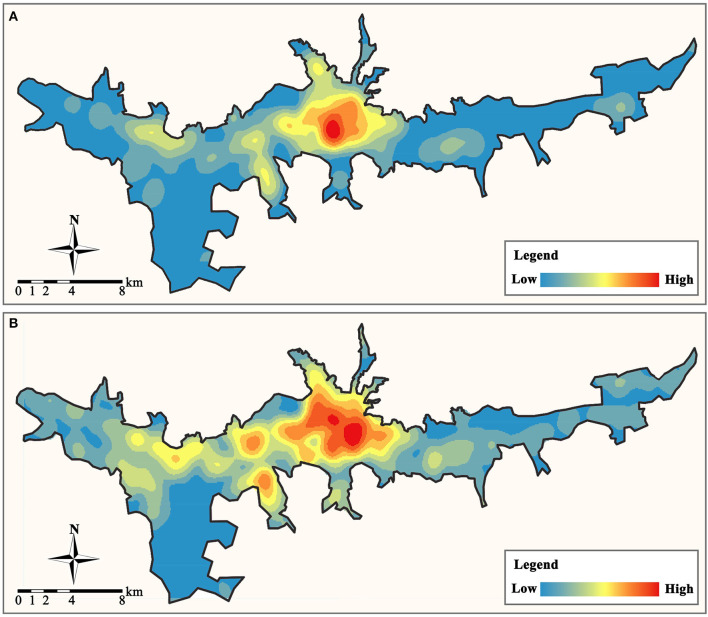
The distribution of primary care facilities and residential locations. **(A)** Primary care facilities. **(B)** Residences.

We compiled 1,040 residences (containing 746 residential communities and 294 villages) and 253 primary care facilities (including 212 community clinics and 41 health centers) in the study area using the POI data. In addition, we gathered data from ten general hospitals as a point of comparison. According to population-based calculations, the total healthcare service supply of community clinics and health centers was 1.06 and 1.23 million, respectively, which could fulfill approximately three times the demand for primary medical services (the total population in the study area was only 0.34 million). Using the GIS kernel density analysis tool, we assessed the current primary care facility locations and residences in the study area. As depicted in Figure 4, the distribution of primary care facilities in Lhasa is comparable with the distribution of residences, with large concentrations in the center and low concentrations in the east and west.

**Figure 5:**
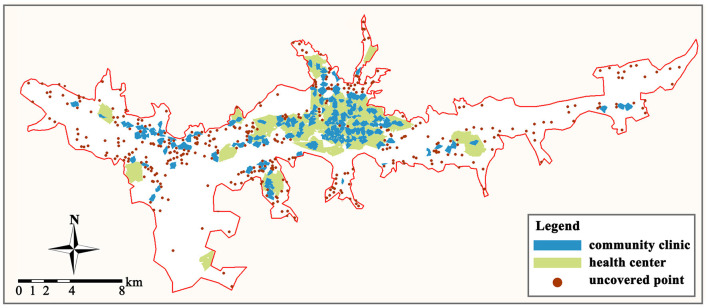
Service area of primary care facilities and uncovered residences.

By creating the service areas of primary care facilities in the GIS platform, the scope and coverage of all facility service areas may be obtained (Figure 5). Overall, only 59.23% of the residences in the study area (616 out of 1,040 places) are covered by the service areas of primary care facilities. And this percentage rises to 77.31% (804) when the service areas of general hospitals are considered. We can see that these unserved communities are primarily located on the eastern, western, and northern land borders of the research area, with primary care facility services being especially scarce in the eastern region.

The Origin-Destination matrix in GIS network analysis was utilized to calculate the actual distance between each residence and the nearest primary care facility and to build their connection. The results of the calculations (Table 1) indicated that the average distance between residences and the nearest care facility exceeded their service radius, and the number of residences within the service area of either community clinics or health centers was less than half of the total. Since the healthcare service duties of community clinics and health centers are not interchangeable, this information is a more accurate indicator of the paucity of primary care facilities than the coverage rate. The community clinic with the highest service pressure would provide medical services to 19 residences (nine of which are inside the service area), compared to 112 for health centers (28 of which are within the service area).

**Table 1:** Results of the Origin-Destination matrix calculation.

**Facilities**	**O-D Distance (m)**			**Sites in the service area**	**Max served**	**Max served (In service area)**
	**Max**	**Min**	**Average**			
Community clinics	4186.2	4.4	636.8	366	19	9
Health centers	7732.7	2.1	1797.4	486	112	28

**Figure 6:**
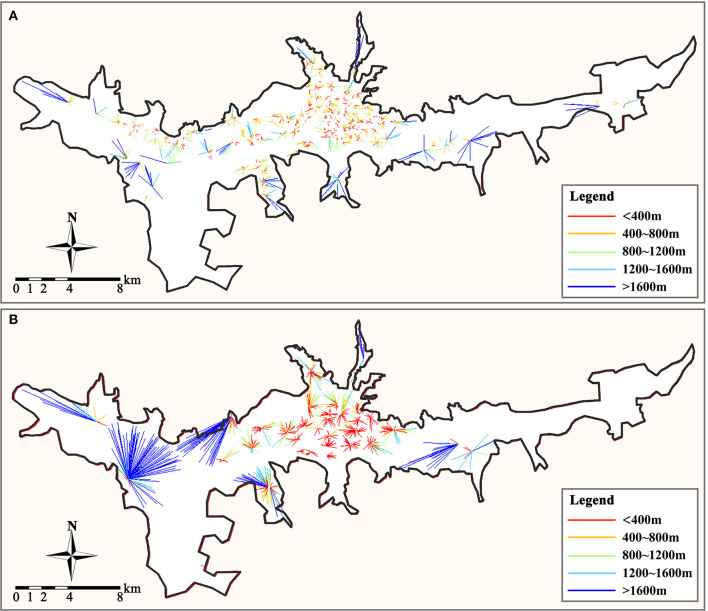
Spatial distribution of Origin-Destination links. **(A)** From residences to community clinic. **(B)** From residences to health center.

**Figure 7:**
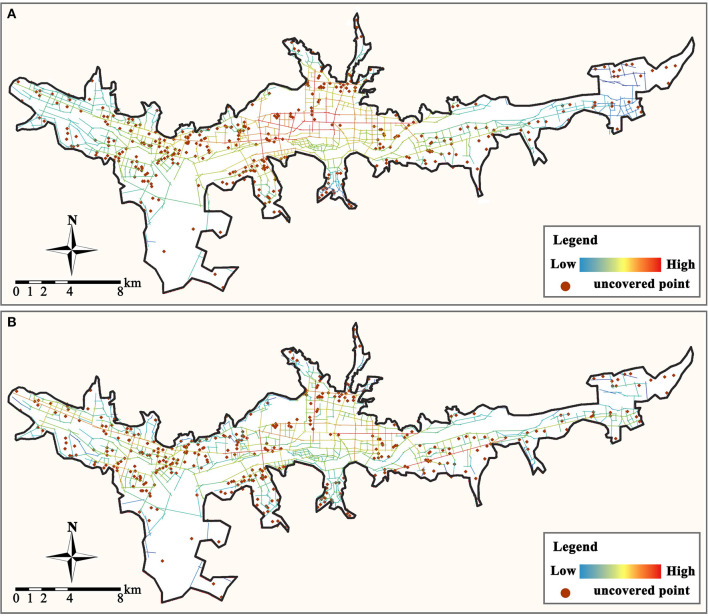
Analysis of urban road axis integration. **(A)** Global integration. **(B)** Local integration.

After visualizing the spatial distribution of the Origin-Destination matrix between the residence and primary care facility (Figure 6), it was discovered that most residences, including those in the east and west areas, have access to a community clinic within half the service radius (400–800 m), whereas health centers are not easily accessible. Most residences at the perimeter of the city's built-up area are more than 3,000 m away from the nearest health center. As a result, residents may not be able to walk to the health center. Therefore, they are required to drive to the general hospital. This can also be independently confirmed by integrating the Depthmap platform integration analysis (Figure 7). Most residences that are not within the service area of primary care facilities are in areas with low global integration, and the local integration with the surrounding three-step topological distance nodes is also significantly insufficient, making it extremely difficult to walk to the care facilities.

**Figure 8:**
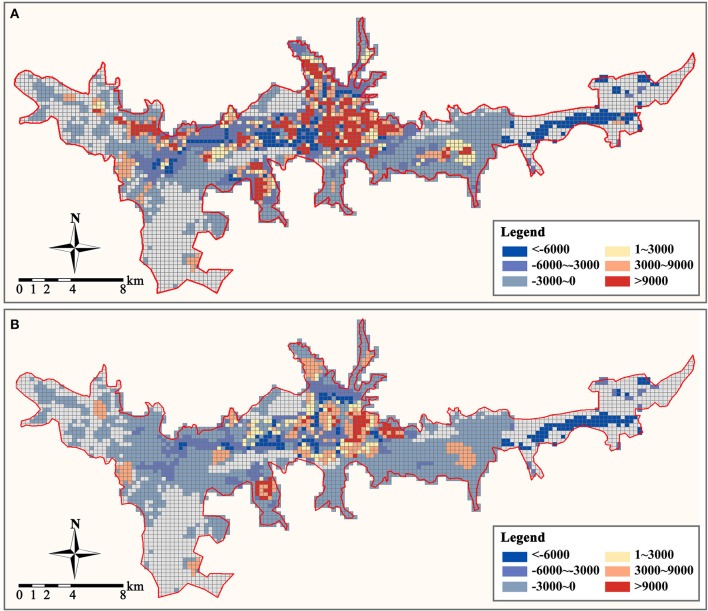
Supply and demand disparity of primary care facilities (person/km2). **(A)** Community clinic. **(B)** Health center.

Finally, we established a 300 × 300 m grid to determine the demand density and supply density of primary care facilities in the study area, where the demand density is the resident population density, and the supply density is the service density of all primary care facilities. We can determine the supply and demand relationship of primary care services in the study area by comparing the disparity between the two to the average demand density in the study area (3,000 people/km^2^ is calculated to be the average demand density in the study region). When the disparity between supply and demand is less than zero, it indicates that regional primary care facilities are inadequately supplied and that residents' demand for medical services is unmet. When the disparity is between (0, 1), it indicates that regional medical service facilities have a balanced supply and demand relationship. When between (1, 3), it indicates that there is an excess of regional primary care facilities. When the disparity exceeds three, primary care services in a region have reached saturation. Our analysis results are depicted in Figure 8. Overall, the distribution of primary care supply and demand in the study area is extremely unbalanced, with community clinics in the central area being severely saturated with supply and health centers supplying at a level that can meet demand. But the level of primary care supply in most areas to the east and west of the study area is insufficient to meet the demand for healthcare services, and there is a severe shortage of primary care facilities.

## Discussion

### Redundancy

In the context of the idea of resilience, redundancy refers to the diversity of functional components and the replicability of functions in a city. It guarantees that if the capacity of a component or a level is compromised, the city system can still rely on other levels to function normally (90, 91). Kharrazi et al. (92) argue that systems can be made more fault-tolerant by replicating and creating new paths, functions, or components, and demonstrate the importance of diversity and redundancy by showing the relationship between modules, nodes, and paths. When the urban healthcare system is confronted with a public health event shock, a large number of seriously ill patients tend to quickly deplete the medical resources of the urban general hospital. Consequently, it is necessary to ensure that the urban primary care facilities have sufficient resources and capacity to meet the requirements for the admission and treatment of mildly ill patients, community governance, and other daily medical services to ensure that the urban healthcare system can withstand the public health event shock.

The total supply of primary healthcare services in the built-up area of Lhasa exceeds the total demand, but the service area of the facility can only cover < 60% of the population. And the primary medical system is still in an imperfect and unbalanced state. In general, people can satisfy their healthcare needs by going to facilities or general hospitals in neighboring communities to avoid “difficulty in seeing a doctor. In general, individuals can avoid “difficult access” by visiting nearby community centers or general hospitals. However, several studies have demonstrated that when a pandemic exacerbates the problem of resource shortages and poor transportation, the healthcare system is overwhelmed (93, 94). When a major public health event such as COVID-19 occurs, nearly half of the communities in the built-up area will have trouble obtaining healthcare services from nearby primary care facilities and will need to deploy personnel and resources from other communities or general hospitals. This will undoubtedly pose a significant challenge to the operational capacity of the urban healthcare system and will indirectly increase the pressure on community governance. When there are no primary care facilities nearby to provide treatment and healthcare services, residents will be forced to travel to health centers or general hospitals further away, which will result in staggering movement within the city, thereby increasing the risk of illness among residents. In contrast, the lack of primary care facilities makes it difficult for community governors to detect and comprehend the health status of inhabitants, hence increasing the likelihood of mass infection. Therefore, it is necessary to increase the redundancy of urban primary care by optimizing the spatial distribution, expanding the service coverage area of primary care facilities, and closing the gap in medical services. This is the fundamental objective for reducing the risk of primary care and bolstering the resilience of urban healthcare in mountainous regions.

### Accessibility

The notion of accessibility is derived from the field of transportation, where it refers to the capacity of individuals to reach a certain place, which is typically expressed by the journey time, cost, number of destinations, and attractiveness between the origin and destination (68, 70–72). Accessibility corresponds to the rapidity in urban resilience characteristics, and a healthcare service system with better overall accessibility can help people access healthcare resources in a timely manner, thereby avoiding or reducing urban risks from public health events (70). And in the recovery phase following a public health event shock, a healthcare facility with high accessibility can also provide more convenient services. Depending on the variation in the function of primary care facilities, national studies have evaluated their accessibility in different ways. As Luo and Wang (66) define the physician's service area by threshold travel time and consider physician availability based on the demand around them in Chicago, while accessibility studies for cities such as Shanghai and Wuhan in China mostly used healthcare facilities as destinations (95, 96). Accessibility encompasses some transportation modes, with pedestrian transportation having the strongest association with primary care facility accessibility.

There is a substantial disparity between the walkability of community clinics and health centers in the primary care facility system in the Lhasa built-up area. Comparatively, only about one-third of the residences have Origin-Destination distances smaller than the service range of community clinics, but the majority do not exceed the service range by too much. This means that when there is no community health station in the community, residents can reach other community care facilities by walking 500 to 1,500 m. However, the accessibility distribution of health centers has some hidden problems. While the Origin-Destination distance to health centers in the middle of the city is within the service area, it is difficult for residents of urban fringe areas to walk to the nearest community health center. And most health centers in urban fringe areas must serve the healthcare needs of 10 to 15 residences, which makes it challenging to match their supply.

### Balance

The equilibrium of public service facilities is manifested on the one hand by the equilibrium between the supply capacity and demand level of healthcare services in a particular area, and on the other hand by the spatial distribution of the supply-demand relationship between different areas within the study's scope (71, 97, 98). The balance of supply and demand is intended to be the equilibrium between all products and services provided by society and social requirements in each period, as evidenced by the changes in product supply and social demands in response to product price changes, and at a certain price point (99, 100). Healthcare services are public services under government regulation and do not fully follow the price-driven market law, but primary care facilities (especially community clinics) are not all publicly owned. Some of them are operated by communities or private contractors, leading to an unbalanced distribution of primary care facilities. This has resulted in an imbalanced urban geographic distribution of primary care services.

The distribution of primary care facilities is crucial for sustaining community resilience. A balanced link between supply-demand and spatial distribution is favorable to macroeconomic regulation of the distribution and use of medical supplies, as well as to the maintenance of community stability and the decrease of crowds during public health events. In densely populated areas with permanent residents, to avoid medical congestion, the supply of primary medical services can be made greater than the residents' medical demand, and multiple points can be dispersed. In urban fringe areas with scattered residential points, consideration must be given to maximizing the service area coverage of primary care facilities to maintain the efficiency of primary care access when there are fewer facilities, and the latent demand is greater. However, the current distribution of primary care facilities at both levels in Lhasa cannot meet the needs of services in densely populated and dispersed areas, and the supply of primary care services in the central area is oversaturated, whereas the majority of areas on the city's periphery have an excess of healthcare services.

### Optimization based on the location-allocation model

As revealed above, the supply-demand relationship and the spatial distribution of primary care facilities in the built-up area of Lhasa are flawed in terms of redundancy, accessibility, and lack of balance. In this study, we optimize the spatial distribution of primary care facilities from a macroscopic perspective to improve the supply-demand relationship of primary medical services while keeping the total supply of primary medical services constant. The maximized coverage model intuitively extends the coverage area of primary care services, reduces the average time for residents to reach primary care facilities, and decreases the difficulty of access to care in areas lacking primary care services. The optimized distribution scheme improves the overall efficiency of the healthcare system and effectively reduces the risks associated with healthcare crowding and resource imbalance, thereby enhancing urban public health resilience.

**Figure 9:**
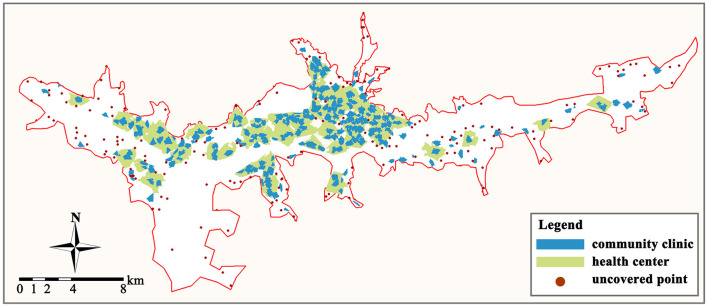
Optimized primary care facility service area and uncovered residences.

The optimized primary care facility system consists of 177 added facility sites (including 152 community clinics and 24 health centers) and 176 retained facility sites (including 60 community clinics and 17 health centers). We reevaluated the supply and demand of optimized primary care facilities based on three indicators: redundancy, accessibility, and balance, to confirm the rationality of the optimized structure of the facilities. Through distribution optimization, the residential point coverage rate of primary care facilities grew from 59.23 to 83.27%, and the capacity of primary care services increased significantly, but there were still some marginal locations that were not covered by the service area (Figure 9). This finding indicates that even with the maximum service area, the current number of primary care facilities cannot cover all residential points in the study area. Therefore, the government needs to appropriately increase the number of primary care facilities according to the fluctuation of healthcare demand or open mobile healthcare service stations in residences that are far from primary care service areas.

**Figure 10:**
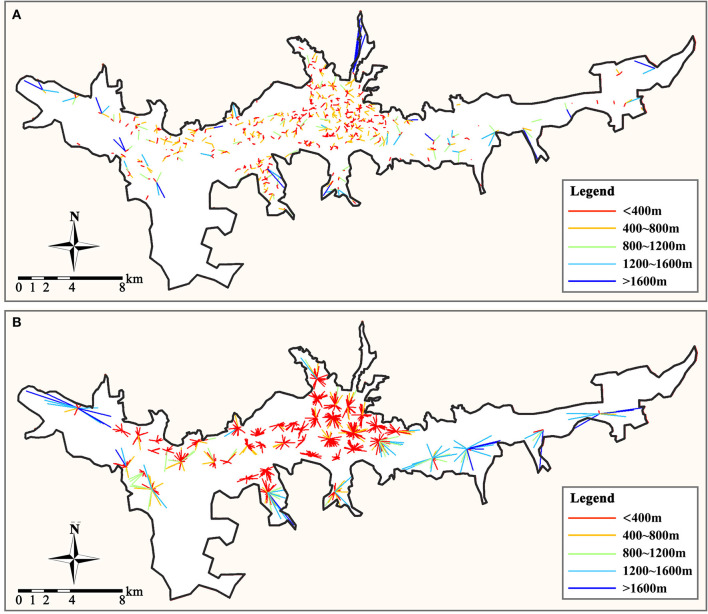
Optimized spatial distribution of Origin-Destination links. **(A)** From residences to community clinic. **(B)** From residences to health center.

After distribution optimization, the average distance between residences and the community clinics is reduced from 636.8 to 364.3 m, and it is reduced from 1977.4 to 912.3 m between residences and health centers. It indicates that the accessibility of primary care facilities is significantly improved. Simultaneously, the maximum number of residences served by community clinics and health centers was reduced from 19 to 11 and 112 to 57, and the demand for primary care services was balanced. Analyzed from the spatial distribution perspective (Figure 10), after distribution optimization, the accessibility of residences to primary care facilities is significantly enhanced, and most residences can quickly reach the nearest facility site. However, there are still some urban fringe areas where residents' accessibility is poor.

**Figure 11:**
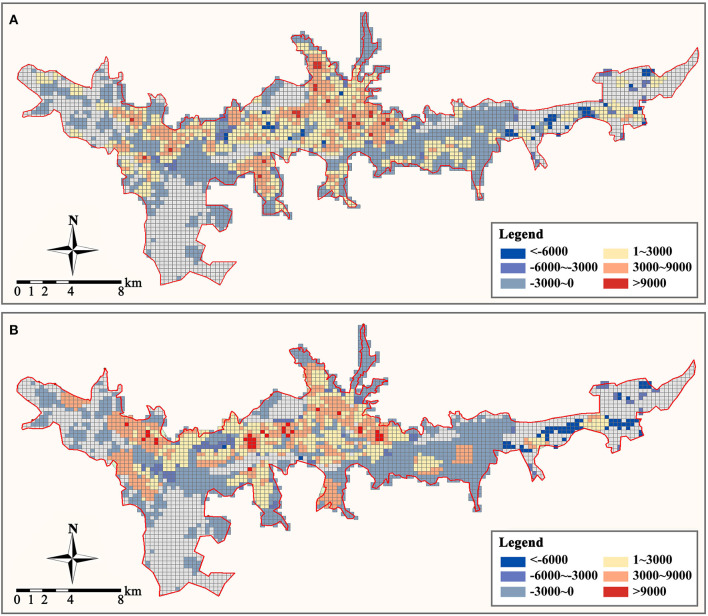
Optimized supply and demand disparity of primary care facilities (person/km^2^). **(A)** Community clinic. **(B)** Health center.

To determine the effect of distribution optimization on the spatial distribution of the primary care supply-demand relationship in the study area, we evaluated the supply-demand density disparity of primary care services within each raster after distribution optimization (Figure 11). After optimization, the overabundance of medical supply in the central area and the severe deficiency of medical supply in the urban periphery can be eased. In the majority of Lhasa's central area, the disparity between primary medical supply and demand is kept between 0 and 3 times the average demand density, which is sufficient to cover both daily and emergency medical demands. Compared to the current arrangement of primary care facilities, the optimal architecture has partially reversed the primary care deficit in most locations on the west and east sides of the research area, although there are still certain areas where primary care services are few. In conducting the residence coverage analysis and constructing the zone distribution model, we use POI points to represent the spatial distribution of each residence. However, the areas of the residences represented by these points vary in size, making it challenging for some communities and villages with large areas and dispersed populations to be covered by primary care services. For these residences, access to the nearest primary care facilities (even if they are slightly outside the service area) via internal roads is still the most logical option.

## Conclusion

This paper employs a GIS-based method to evaluate the supply-demand relationship and spatial distribution rationality of urban primary care facilities in the built-up area of Lhasa, China, as the study area to conduct an empirical study and proposes an optimization scheme for the spatial distribution of primary care facilities in conjunction with a location-allocation model. The program may effectively alleviate the oversupply and uneven distribution of primary care facilities in the study area, allowing them to satisfy the development needs of public health resilience and withstand the effects of disasters.

This paper's findings, methodology, and technique have practical consequences for the site development of urban healthcare facilities in mountainous cities. Firstly, this paper integrates the concept of urban resilience to comprehensively assess and optimize the spatial distribution of urban primary care facilities, so that primary medical services can not only meet the daily needs of residents but also withstand the impact of public health events on the urban medical system. Secondly, existing studies are less involved in the underdeveloped regions of the highland area, which have lagging economic development. This paper integrates healthcare demand into the study of the spatial distribution of primary care facilities and establishes an assessment system combining redundancy, accessibility, and balance from the perspective of supply-demand balance, providing a new idea for the existing care facility distribution assessment research. At last, this paper presents a maximum coverage model that maintains the total amount of amenities, which can result in a more acceptable distribution optimization strategy for highland cities. This paper presents the importance of the distribution of primary care facilities. The results of the study can optimize and complement the existing hierarchical healthcare system to help build a more rational primary care network in highland areas and other underdeveloped regions, thereby reducing urban healthcare risks and enhancing urban public health resilience.

Nonetheless, the study does have certain drawbacks. Through analysis and evaluation, it is evident that the spatial distribution of primary care facilities in the study area is unbalanced. This imbalance is reflected in the number and supply of facilities as well as spatial accessibility. There are differences in the spatial distribution characteristics of facilities at various levels, and these problems are the result of a combination of factors. Not only is the distribution of healthcare services and function of supply and demand, but also the placement and distribution of geographic places. The optimization scheme based just on population, facility distribution, and road traffic is insufficient. It must also consider the influence of regional economics, policies, external transportation, ecological environment, geography, and other public service facilities siting. In addition, the demand for primary care services may be different for each population group, and the redundancy and accessibility of facilities are correlated with the population characteristics of the surrounding residences, thus affecting the study results. Therefore, we believe that it is possible to evaluate the distribution of urban care facilities from more perspectives and to superimpose the evaluation results from different perspectives, which will make the evaluation and optimization results of the spatial distribution of care facilities more scientific and guiding. It will be widely applicable to other site selection and distribution studies.

## Data availability statement

The raw data supporting the conclusions of this article will be made available by the authors, without undue reservation.

## Author contributions

YY: conceptualization, funding acquisition, and supervision. RZ: formal analysis, methodology, and writing. LQ, XY, LD, and GZ: review and editing. All authors contributed to the article and approved the submitted version.

## References

[1] Wong CY Wang IK Sheu J Hu MC. What network orientation supports the development of a Resilient City? evidence from the innovation systems of eighty-seven cities. Cities. (2022) 131:103923. 10.1016/j.cities.2022.103923

[2] Feng Y Lee CC Peng D. Does regional integration improve economic resilience? Evidence from urban agglomerations in China. Sustain Cities Soc. (2022) 88:104273. 10.1016/j.scs.2022.104273

[3] Maclean K Cuthill M Ross H. Six attributes of social resilience. J Environ Plan Manag. (2014) 57:144–56. 10.1080/09640568.2013.763774

[4] Perrings C. Resilience and sustainable development. Environ Dev Econ. (2006) 11:417–27. 10.1017/S1355770X06003020

[5] Pickett STA McGrath B Cadenasso ML Felson AJ. Ecological resilience and resilient cities. Build Res Inf. (2014) 42:143–57. 10.1080/09613218.2014.850600

[6] Liu D Sun T Liu D Fu Q Zhang L Li X. A new method to analyze the driving mechanism of flood disaster resilience and its management decision-making. J Hydrol. (2022) 612:128134. 10.1016/j.jhydrol.2022.128134

[7] Murgatroyd A Hall JW. The resilience of inter-basin transfers to severe droughts with changing spatial characteristics. Front Environ Sci. (2020) 8:571647. 10.3389/fenvs.2020.571647

[8] Valdés HM Amaratunga D Haigh R. Making cities resilient: from awareness to implementation. Int J Disaster Resil Built Environ. (2013) 4:5–8. 10.1108/17595901311299035

[9] Manyena SB. The concept of resilience revisited. Disasters. (2006) 30:434–50. 10.1111/j.0361-3666.2006.00331.x 17100752

[10] Vale LJ Campanella TJ. The Resilient City: How Modern Cities Recover From Disaster. New York, NY: Oxford University Press (2005). p. 376. 10.1093/oso/9780195175844.001.0001

[11] Wang L. Exploring a knowledge map for urban resilience to climate change. Cities. (2022) 131:104048. 10.1016/j.cities.2022.104048

[12] Sabatino M. Economic crisis and resilience: Resilient capacity and competitiveness of the enterprises. J Bus Res. (2016) 69:1924–924. 10.1016/j.jbusres.2015.10.081

[13] Cheng T Zhao Y Zhao C. Exploring the spatio-temporal evolution of economic resilience in Chinese cities during the COVID-19 crisis. Sustain Cities Soc. (2022) 84:103997. 10.1016/j.scs.2022.103997

[14] Umakanthan S Bukelo MM Gajula SS. The commonwealth Caribbean COVID-19: regions resilient pathway during pandemic. Front Public Health. (2022) 10:844333. 10.3389/fpubh.2022.844333 35664108 PMC9160791

[15] Banai R. Pandemic and the planning of resilient cities and regions. Cities. (2020) 106:102929. 10.1016/j.cities.2020.102929 32952256 PMC7490286

[16] Wu C Cenci J Wang W Zhang JZ. Resilient city: characterization, challenges and outlooks. Buildings. (2022) 12:516. 10.3390/buildings12050516

[17] Orsetti E Tollin N Lehmann M Valderrama VA MoratM J. Building resilient cities: climate change and health interlinkages in the planning of public spaces. Int J Environ Res Public Health. (2022) 19:1355. 10.3390/ijerph19031355 35162378 PMC8835258

[18] Bhaskar S Bradley S Chattu VK Adisesh A Nurtazina A Kyrykbayeva S. Telemedicine across the globe-position paper from the COVID-19 pandemic health system resilience PROGRAM (REPROGRAM) international consortium (Part 1). Front Public Health. (2020) 8:556720. 10.3389/fpubh.2020.556720 33178656 PMC7596287

[19] Marmo R Pascale F Diana L Sicignano E Polverino F. Lessons learnt for enhancing hospital resilience to pandemics: a qualitative analysis from Italy. Int J Disaster Risk Reduct. (2022) 81:103265. 10.1016/j.ijdrr.2022.103265 36061241 PMC9419438

[20] Liu Z Ma R Wang HJ. Assessing urban resilience to public health disaster using the rough analytic hierarchy process method: a regional study in China. J Safety Sci Resil. (2022) 3:93–104. 10.1016/j.jnlssr.2021.12.003 PMC9671553 40478116

[21] Grimaz S Ruzzene E Zorzini F. Situational assessment of hospital facilities for modernization purposes and resilience improvement. Int J Disaster Risk Reduct. (2021) 66:102594. 10.1016/j.ijdrr.2021.102594 34567962 PMC8452357

[22] Li L Liao S Yuan J Wang E She J. Analyzing healthcare facility resilience: scientometric review and knowledge map. Front Public Health. (2021) 9:764069. 10.3389/fpubh.2021.764069 34820352 PMC8606559

[23] World Health Organization. Integrating Health Services. Technical Series on Primary Healthcare. Geneva: World Health Organization (2018). Available online at: https://www.who.int/docs/default-source/primary-health-care-conference/linkages.pdf?sfvrsn=bfbb4059_2 (accessed December 30, 2022).

[24] Del Conte DE Locascio A Amoruso J McNamala ML. Modeling multimodal access to primary care in an urban environment. Transp Res Interdiscip Perspect. (2022) 13:100550. 10.1016/j.trip.2022.100550

[25] Kinder K Bazemore A Taylor M Mannie C Strydom S George J. Integrating primary care and public health to enhance response to a pandemic. Prim Healthcare Res Dev. (2021) 22:e27. 10.1017/S1463423621000311 34109936 PMC8220344

[26] Leslie M Fadaak R Pinto N Davies J Green L Seidel J. Achieving resilience in primary care during the COVID-19 pandemic: competing visions and lessons from Alberta. Healthcare Policy. (2021) 17:54. 10.12927/hcpol.2021.26657 34895410 PMC8665729

[27] Starfield B Shi L Macinko J. Contribution of primary care to health systems and health. Milbank Q. (2005) 83:457–502. 10.1111/j.1468-0009.2005.00409.x 16202000 PMC2690145

[28] Aghapour AH Yazdani M Jolai F Mojtahedi M. Capacity planning and reconfiguration for disaster-resilient health infrastructure. J Build Eng. (2019) 26:100853. 10.1016/j.jobe.2019.100853

[29] Hu P Liu Z Lan J. Equity and efficiency in spatial distribution of basic public health facilities: a case study from Nanjing metropolitan area. Urban Policy Res. (2018) 37:243–66. 10.1080/08111146.2018.1523055

[30] Pearce J Witten K Bartie P. Neighbourhoods and health: a GIS approach to measuring community resource accessibility. J Epidemiol Community Health. (2006) 60:389–95. 10.1136/jech.2005.043281 16614327 PMC2563982

[31] Holling CS. Resilience and stability of ecological systems. Annu Rev Ecol Syst. (1973) 4:1–23. 10.1146/annurev.es.04.110173.000245

[32] Wang CH Blackmore JM. Resilience concepts for water resource systems. J Water Resour Plann Manag. (2009) 135:528–36. 10.1061/(ASCE)0733-9496(2009)135:6(528)

[33] Liao KH. A theory on urban resilience to floods—a basis for alternative planning practices. Ecol Soc. (2012) 17:48. 10.5751/ES-05231-170448

[34] Walker B Holling CS Carpenter SR Kinzig A. Resilience, adaptability and transformability in social-ecological systems. Ecol Soc. (2004) 9:5. 10.5751/ES-00650-090205

[35] Folke C. Resilience: The emergence of a perspective for socialrmability in social-ecological*Global Environ Change*. (2006) 16:253–67. 10.1016/j.gloenvcha.2006.04.002

[36] Mou Y Luo Y Su Z Wang J Liu T. Evaluating the dynamic sustainability and resilience of a hybrid urban system: case of Chengdu, China. J Clean Prod. (2021) 291:125719. 10.1016/j.jclepro.2020.125719

[37] Brown A Dayal A Rumbaitis Del Rio C. From practice to theory: emerging lessons from Asia for building urban climate change resilience. Environ Urban. (2012) 24:531–56. 10.1177/0956247812456490

[38] Zhao R Fang C Liu J Zhang L. The evaluation and obstacle analysis of urban resilience from the multidimensional perspective in Chinese cities. Sustain Cities Soc. (2022) 86:104160. 10.1016/j.scs.2022.104160

[39] Pickett STA Cadenasso ML Grove JM. Resilient cities: meaning, models, and metaphor for integrating the ecological, socio-economic, and planning realms. Landsc Urban Plan. (2004) 69:369–84. 10.1016/j.landurbplan.2003.10.035

[40] Ahern J. From fail-safe to safe-to-fail: sustainability and resilience in the new urban world. Landsc Urban Plan. (2011) 100:341–3. 10.1016/j.landurbplan.2011.02.021

[41] Jha AK Todd WM Stanton-Geddes Z. Building Urban Resilience: Principles, Tools, and Practice. World Bank Publications (2013). p. 182. 10.1596/978-0-8213-8865-5

[42] Boyd E Folke C. Adapting Institutions: Governance, Complexity and Social-Ecological Resilience. Cambridge: Cambridge University Press (2011). p. 290. 10.1017/CBO9781139017237

[43] Jabareen Y. Planning the resilient city: concepts and strategies for coping with climate change and environmental risk. Cities. (2013) 31:220–9. 10.1016/j.cities.2012.05.004

[44] Wilkinson C. Social-ecological resilience: insights and issues for planning theory. Planning Ther. (2012) 11:148–69. 10.1177/1473095211426274

[45] Kruk ME Ling EJ Bitton A Cammett M Cavanaugh K Chopra M. Building resilient health systems: a proposal for a resilience index. BMJ. (2017) 357:j2323. 10.1136/bmj.j2323 28536191

[46] Iflaifel M Lim RH Ryan K Crowley C. Resilient healthcare: a systematic review of conceptualisations, study methods and factors that develop resilience. BMC Health Serv Res. (2020) 20:1–21. 10.1186/s12913-020-05208-3 32303209 PMC7165381

[47] Ellis L A Churruca K Clay-Williams R Pomare C Austin EE Long JC. Patterns of resilience: a scoping review and bibliometric analysis of resilient healthcare. Saf Sci. (2019) 118:241–57. 10.1016/j.ssci.2019.04.044

[48] Curtis S Fair A Wistow J Val DV Oven K. Impact of extreme weather events and climate change for health and social care systems. Environ Health. (2017) 16:23–32. 10.1186/s12940-017-0324-3 29219105 PMC5773887

[49] Zhong S Clark M Hou XY Zang YL Fitzgerald G. Development of hospital disaster resilience: conceptual framework and potential measurement. Emerg Med J. (2014) 31:930–8. 10.1136/emermed-2012-202282 24028975

[50] Achour N Price ADF. Resilience strategies of healthcare facilities: present and future. Int J Disaster Resil Built Environ. (2010) 1:264–76. 10.1108/17595901011080869

[51] Kieny M-P Evans DB Schmets G Kadandale S. Health-system resilience: reflections on the Ebola crisis in western Africa. Bull World Health Organ. (2014) 92:850. 10.2471/BLT.14.149278 25552765 PMC4264399

[52] Kruk ME Myers M Varpilah ST Dahn BT. What is a resilient health system? lessons from Ebola. Lancet. (2015) 385:1910–910. 10.1016/S0140-6736(15)60755-3 25987159

[53] Cimellaro GP Marasco S Noori AZ Mahin SA. A first order evaluation of the capacity of a healthcare network under emergency. Earthq Eng Eng Vib. (2019) 18:663–77. 10.1007/s11803-019-0528-3

[54] Hassan EM Mahmoud H. An integrated socio-technical approach for post-earthquake recovery of interdependent healthcare system. Reliabil Eng Syst Saf. (2020) 201:15. 10.1016/j.ress.2020.106953

[55] Ferguson WJ Kemp K Kost G. Using a geographic information system to enhance patient access to point-of-care diagnostics in a limited-resource setting. Int J Health Geogr. (2016) 15:12. 10.1186/s12942-016-0037-9 26932155 PMC4774034

[56] Godschalk DR. Urban hazard mitigation: creating resilient cities. Nat Hazards Rev. (2003) 4:136–43. 10.1061/(ASCE)1527-6988(2003)4:3(136)

[57] Yang L Wang B Zhou J Wang X. Walking accessibility and property prices. Transp Res D: Transp Environ. (2018) 62:551–62. 10.1016/j.trd.2018.04.001

[58] Yang L Chau KW Wang X. Are low-end housing purchasers more willing to pay for access to public services? evidence from China. Res Transp Econ. (2019) 76:100734. 10.1016/j.retrec.2019.06.001

[59] Demitiry M Higgins CD PM A Miller EJ. Accessibility to primary care physicians: comparing floating catchments with a utility-based approach. J Transp Geogr. (2022) 101:103356. 10.1016/j.jtrangeo.2022.103356

[60] Hone T Macinko J Millett C. Revisiting alma-ata: what is the role of primary healthcare in achieving the sustainable development goals? Lancet. (2018) 392:1461–4612. 10.1016/S0140-6736(18)31829-4 30343860

[61] Akman M Ayhan Baaan D Usanma Koban B Marti T Decat P Lefeuvre Y. Organization of primary care. Prim Health Care Res Dev. (2022) 23:e49. 10.1017/S1463423622000275 36047002 PMC9472237

[62] Church RL Murray AT. Business Site Selection, Location Analysis, and GIS. Hoboken, NJ: John Wiley & Sons (2009). p. 306. 10.1002/9780470432761

[63] Liang GS Wang MJJ. A fuzzy multi-criteria decision-making method for facility site selection. Int J Prod Res. (1991) 29:2313–313. 10.1080/0020754910894808519477577

[64] Hansen WG. How accessibility shapes land use. J Am Inst Plann. (1959) 25:73–6. 10.1080/01944365908978307

[65] Talen E Anselin L. Assessing spatial equity: an evaluation of measures of accessibility to public playgrounds. Environ Plann A. (1998) 30:595–613. 10.1068/a300595

[66] Luo W Wang F. Measures of spatial accessibility to healthcare in a GIS environment: synthesis and a case study in the Chicago region. Environ Plann B. (2003) 30:865–84. 10.1068/b29120 34188345 PMC8238135

[67] Apparicio P SP Si AM. Measuring the accessibility of services and facilities for residents of public housing in Montreal. Urban Stud. (2006) 43:187–211. 10.1080/00420980500409334

[68] Moturi AK Suiyanka L Mumo E Snow RW Okiro EA Macharia PM. Geographic accessibility to public and private health facilities in Kenya in 2021: an updated geocoded inventory and spatial analysis. Front Public Health. (2022) 10:4245. 10.3389/fpubh.2022.1002975 36407994 PMC9670107

[69] Jin M Liu L Tong D Gong Y Liu Y. Evaluating the spatial accessibility and distribution balance of multi-level medical service facilities. Int J Environ Res Public Health. (2019) 16:1150. 10.3390/ijerph16071150 30935065 PMC6479551

[70] Wang W Zhou Z Chen J Cheng W Chen J. Analysis of location selection of public service facilities based on urban land accessibility. Int J Environ Res Public Health. (2021) 18:516. 10.3390/ijerph18020516 33435188 PMC7826736

[71] Silva LL Carvalho Dutra AD Andrade LD Iora PH Rodrigues Ramajo GL Peres Gualda IA. Emergency care gap in Brazil: geographical accessibility as a proxy of response capacity to tackle COVID-19. Front Public Health. (2021) 9:740284. 10.3389/fpubh.2021.740284 34869155 PMC8634954

[72] Fu S Liu Y Fang Y. Measuring the differences of public health service facilities and their influencing factors. Land. (2021) 10:1225. 10.3390/land10111225

[73] Daskin MS Maass KL. The *p*-median problem. In:Laporte G Nickel S Saldanha da Gama F, editor. Location science. Cham: Springer (2015). p. 21–45. 10.1007/978-3-319-13111-5_2

[74] White JA Case KE. On covering problems and the central facilities location problem. Geogr Anal. (1974) 6:281–94. 10.1111/j.1538-4632.1974.tb00513.x

[75] Wang F Tang Q. Planning toward equal accessibility to services: a quadratic programming approach. Environ Plann B. (2013) 40:195–212. 10.1068/b37096

[76] Gu W Wang X McGregor SE. Optimization of preventive healthcare facility locations. Int J Health Geogr. (2010) 9:1–16. 10.1186/1476-072X-9-17 20298608 PMC3161374

[77] Drezner Z Wesolowsky GO. Facility location when demand is time dependent. Naval Res Logist. (1991) 38:763–77. 10.1002/1520-6750(199110)38:5<763::AID-NAV3220380510>3.0.CO;2-A

[78] Gregg SR Mulvey J M. Wolpert J. A stochastic planning system for siting and closing public service facilities. Environ Plann A. (1991) 20:83–98. 10.1068/a200083

[79] Teixeira JC Antunes AP. A hierarchical location model for public facility planning. Eur J Oper Res. (2008) 185:92–104. 10.1016/j.ejor.2006.12.027

[80] Song ZN Chen W Che QJ Zhang L. Measurement of spatial accessibility to healthcare facilities and defining health professional shortage areas based on improved potential model—a case study of rudong county in Jiangsu province. Scientia Geographica Sinica. (2010) 30:213–9.

[81] Yang L Yu B Liang Y Lu Y Li W. Time-varying and non-linear associations between metro ridership and the built environment. Tunn Undergr Space Technol. (2023) 132:104931. 10.1016/j.tust.2022.104931

[82] Yang L Liang Y He B Yang H Lin D. COVID-19 moderates the association between to-metro and by-metro accessibility and house prices. Transp Res D Transp Environ. (2023) 114:103571. 10.1016/j.trd.2022.103571

[83] Jiang S Alves A Rodrigues F Ferreira JrJ Pereira FC. Mining point-of-interest data from social networks for urban land use classification and disaggregation. Comput Environ Urban Syst. (2015) 53:36–46. 10.1016/j.compenvurbsys.2014.12.001

[84] Yang L Liang Y He B Lu Y Gou Z. COVID-19 effects on property markets: The pandemic decreases the implicit price of metro accessibility. Tunn Undergr Space Technol. (2022) 125:104528. 10.1016/j.tust.2022.104528

[85] Zhou Z Zhao Y Shen C Lai S Nawaz R Gao J. Evaluating the effect of hierarchical medical system on health seeking behavior: a difference-in-differences analysis in China. Soc Sci Med. (2021) 268:113372. 10.1016/j.socscimed.2020.113372 32979776

[86] Wang J Wang P Wang X Zheng Y Xiao Y. Use and prescription of antibiotics in primary healthcare settings in China. JAMA Intern Med. (2014) 174:1914–9144. 10.1001/jamainternmed.2014.5214 25285394

[87] Wu D Lam TP Lam KF Zhou XD Sun KS. Health reforms in china: the public's choices for first-contact care in urban areas. Fam Pract. (2017) 34:194–200. 10.1093/fampra/cmw133 28122845

[88] Bruneau M Chang SE Eguchi RT Lee GC O'Rourke TD Reinhorn AM. A framework to quantitatively assess and enhance the seismic resilience of communities. Earthq Spectra. (2003) 19:733–52. 10.1193/1.1623497

[89] Johnson C Blackburn S. Advocacy for urban resilience: UNISDRon making cities resilient campaign. Environ Urban. (2014) 26:29–52. 10.1177/0956247813518684

[90] Wardekker JA De Jong A Knoop JM van der Sluijs JP. Operationalising a resilience approach to adapting an urban delta to uncertain climate changes. Technol Forecast Soc Change. (2010) 77:987–98. 10.1016/j.techfore.2009.11.005

[91] Taleai M Sliuzas R Flacke J. An integrated framework to evaluate the equity of urban public facilities using spatial multi-criteria analysis. Cities. (2014) 40:56–69. 10.1016/j.cities.2014.04.006

[92] Kharrazi A Yu Y Jacob A Vora N Fath BD. Redundancy, diversity, and modularity in network resilience: applications for international trade and implications for public policy. Curr Res Environ Sustain. (2020) 2:100006. 10.1016/j.crsust.2020.06.001 34977604 PMC7382575

[93] Yeoh K Wu Y Chakraborty S Elhusseiny G Gondhowiardjo S Joseph N. Global health system resilience during encounters with stressorsapplications for international trade and implica COVID-19 pandemic. Clin Oncol. (2023). 10.1016/j.clon.2023.01.004 PMC9842532 36764875

[94] Paschoalotto MAC Lazzari EA Rocha R Massuda A Castro MC. Health systems resilience: is it time to revisit resilience after COVID-19? Soc Sci Med. (2023) 320:115716. 10.1016/j.socscimed.2023.115716 36702027 PMC9851720

[95] Shen Y Tao Y. Associations between spatial access to medical facilities and health-seeking behaviors: a mixed geographically weighted regression analysis in Shanghai, China. Appl Geograp. (2022) 139:102644. 10.1016/j.apgeog.2022.102644

[96] Xia Y Chen H Zuo C Zhang N. The impact of traffic on equality of urban healthcare service accessibility: a case study in Wuhan, China. Sustain Cities Soc. (2022) 86:104130. 10.1016/j.scs.2022.104130

[97] Zhang D Zhang G Zhou C. Differences in accessibility of public health facilities in hierarchical municipalities and the spatial pattern characteristics of their services in doumen district, China. Land. (2021) 10:1249. 10.3390/land10111249

[98] Coston JM. Administrative avenues to democratic governance: the balance of supply and demand. Public Adm Dev Int J Manag Res Practice. (1998) 18:479–93. 10.1002/(SICI)1099-162X(199812)18:5<479::AID-PAD37>3.0.CO;2-Y

[99] Landry M D Hamdan E Al Mazeedi S Brooks D. The precarious balance between esupplyupplrooks D. The precarious balance between etween etween tween nce between ities in hierarchiividuals living with chronic obstructive pulmonary disease. Int J Chron Obstruct Pulmon Dis. (2008) 3:393. 10.2147/COPD.S3568 18990966 PMC2629990

[100] Guo M Liu B Tian Y Xu D. Equity to urban parks for elderly residents: perspectives of balance between supply and demand. Int J Environ Res Public Health. (2020) 17:8506. 10.3390/ijerph17228506 33212861 PMC7698437

